# Symptomatic jejunal ectopic pancreas presenting as intestinal obstruction: a case report

**DOI:** 10.1093/jscr/rjaf645

**Published:** 2025-08-23

**Authors:** Abhiraj Yadav, Shreya Muddana, Neha Arutla, Noorudin Ansari

**Affiliations:** Department of General Surgery, Manipal College of Medical Sciences, Pokhara 33700, Kaski, Nepal; Department of General Surgery, Mamata Academy of Medical Sciences, Hyderabad 500118, India; Department of General Surgery, Mamata Academy of Medical Sciences, Hyderabad 500118, India; Department of General Surgery, Mamata Academy of Medical Sciences, Hyderabad 500118, India

**Keywords:** ectopic pancreas, intestinal obstruction, jejunum, heterotopia, case report, histopathology

## Abstract

Ectopic pancreas is a rare condition where pancreatic tissue develops outside its normal location, often remaining asymptomatic. We present a case of a 45-year-old woman with type 2 diabetes and hypertension who presented with postprandial abdominal pain, projectile vomiting, and constipation, clinically suggestive of intestinal obstruction. Computed tomography revealed jejunal wall thickening, raising suspicion of a neoplastic lesion. Exploratory laparotomy identified a 4 cm sessile mass in the jejunum, which was resected. Histopathology confirmed ectopic pancreatic tissue (Type I) with acini, ducts, and islets in the submucosa and muscularis, causing luminal narrowing. The patient recovered well after surgery, with complete symptom resolution. This case highlights ectopic pancreas as a rare but important differential for small bowel obstruction, requiring histopathological confirmation for definitive diagnosis. Surgical resection remains the treatment of choice for symptomatic cases.

## Introduction

Ectopic pancreas is defined as the presence of pancreatic tissue outside its typical location and is a fairly uncommon anomaly, with an incidence of 0.5%–13.7% in autopsy findings in the general population [1]. The incidence of ectopic pancreas in living population is difficult to determine as most patients are asymptomatic [2]. This rare congenital anomaly usually occurs in the upper gastrointestine tract, with the stomach being the most common site; other sites include the spleen, appendix, omentum, gallbladder, fallopian tubes, lungs, or mediastinum [3]. We present you a case of jejunal ectopic pancreas in a mid-aged adult female who presented symptomatically as intestinal obstruction.

## Case history

A 45-year-old female presented to the general surgery outpatient department with complaints of recurrent postprandial abdominal pain, projectile vomiting, and constipation for the past 2 days. The pain was described as crampy, localized to the umbilical region, insidious in onset, and non-radiating. Initially, the pain was mild and intermittent but had progressively worsened, becoming more intense and persistent, associated with bloating, particularly after meals. The vomiting began on the same day as the abdominal pain occurring after meals which was forceful and projectile containing partially digested food, non-bilious, non-bloody occurring multiple times a day. She also experienced constipation with no passage of stool or flatus. She had no bowel movements for 48 h and reported a sensation of incomplete evacuation.

There was no history of jaundice, dyspepsia, weight loss, hematemesis, melena, or any other systemic symptoms.

The patient had a known history of type 2 diabetes mellitus for the past 3 years, managed with oral hypoglycemic agents. She had also been hypertensive for the past 3 years and was on regular antihypertensive medication. She had undergone a hysterectomy for uterine fibroids 6 months prior. The patient has no history of smoking or alcohol use. She follows a mixed diet and has experienced a reduced appetite. There was no significant family history.

### Abdominal examination

On inspection, the abdomen was soft and non-distended, with no visible peristalsis or swelling. A lower midline scar was present, indicative of a previous hysterectomy. On palpation, tenderness was present in the umbilical region without rebound tenderness, guarding or rigidity. Fullness was noted in the left iliac fossa, along with scar tenderness. On auscultation, hyperactive bowel sounds (+++) were noted.

**Figure 1:**
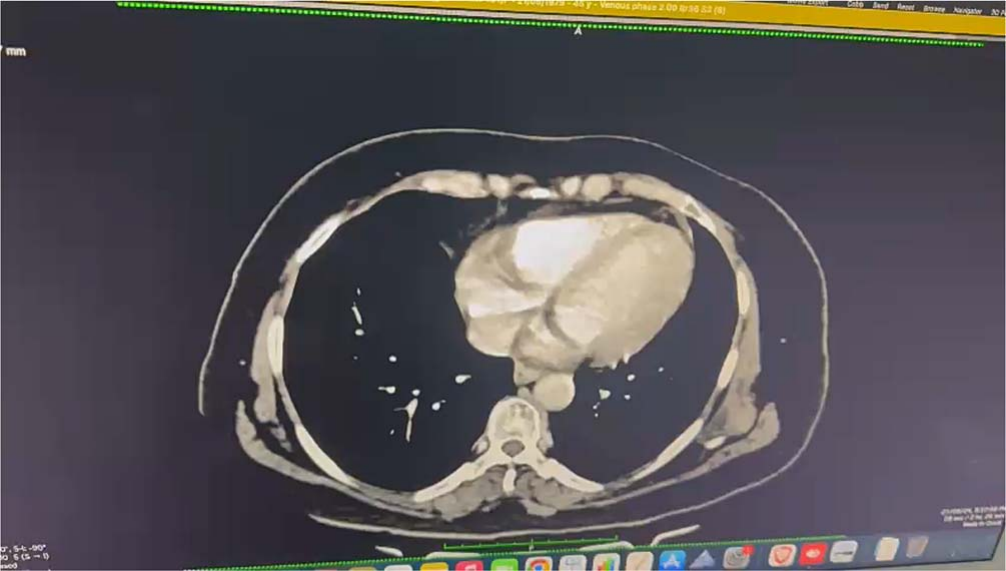
Axial CECT image shows a short segment of asymmetric enhancing wall thickening involving the proximal to mid-jejunal loop (8 cm). There is luminal narrowing at the site of thickening, with adjacent fat stranding and mesenteric lymphadenopathy.

**Figure 2:**
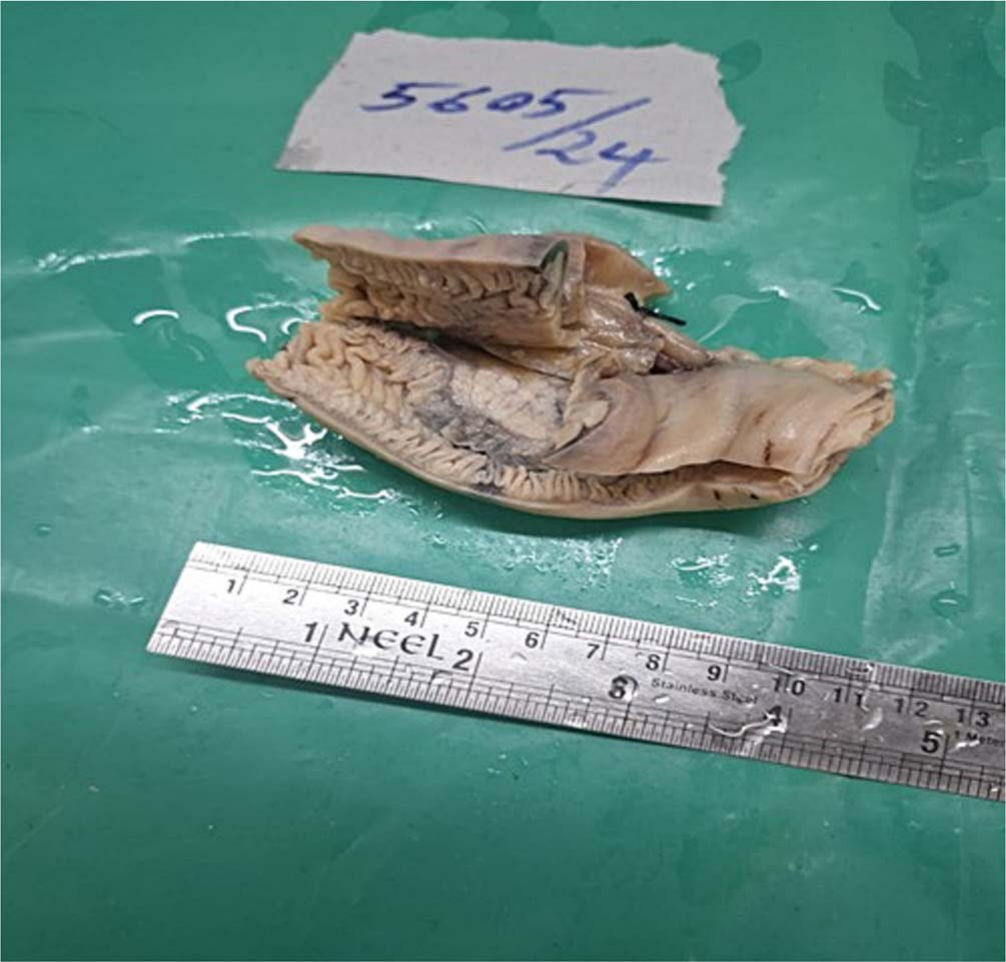
Resected specimen of jejunum measuring ⁓10 cm in length. External surface shows a sessile polypoid mass measuring (4 × 3 cm).Cut surface reveals multiple small, gray–white, speckled areas corresponding to the underlying lesion. The mucosal surface overlying the mass is ulcerated and shows a sessile protrusion (3.5 × 2 cm).

A provisional diagnosis of subacute intestinal obstruction likely secondary to an unusual etiology and further investigations were ordered. Contrast enhanced computed tomography (CECT) of the abdomen revealed short segments of symmetrically thickened walls in the proximal to mid-jejunal loop, accompanied by luminal narrowing, adjacent fat stranding, and mesenteric lymph nodes, raising suspicion of a neoplastic etiology (Fig. 1). An exploratory laparotomy was performed, during which a segment of the jejunum was resected for histopathological examination (Fig. 2).

**Figure 3:**
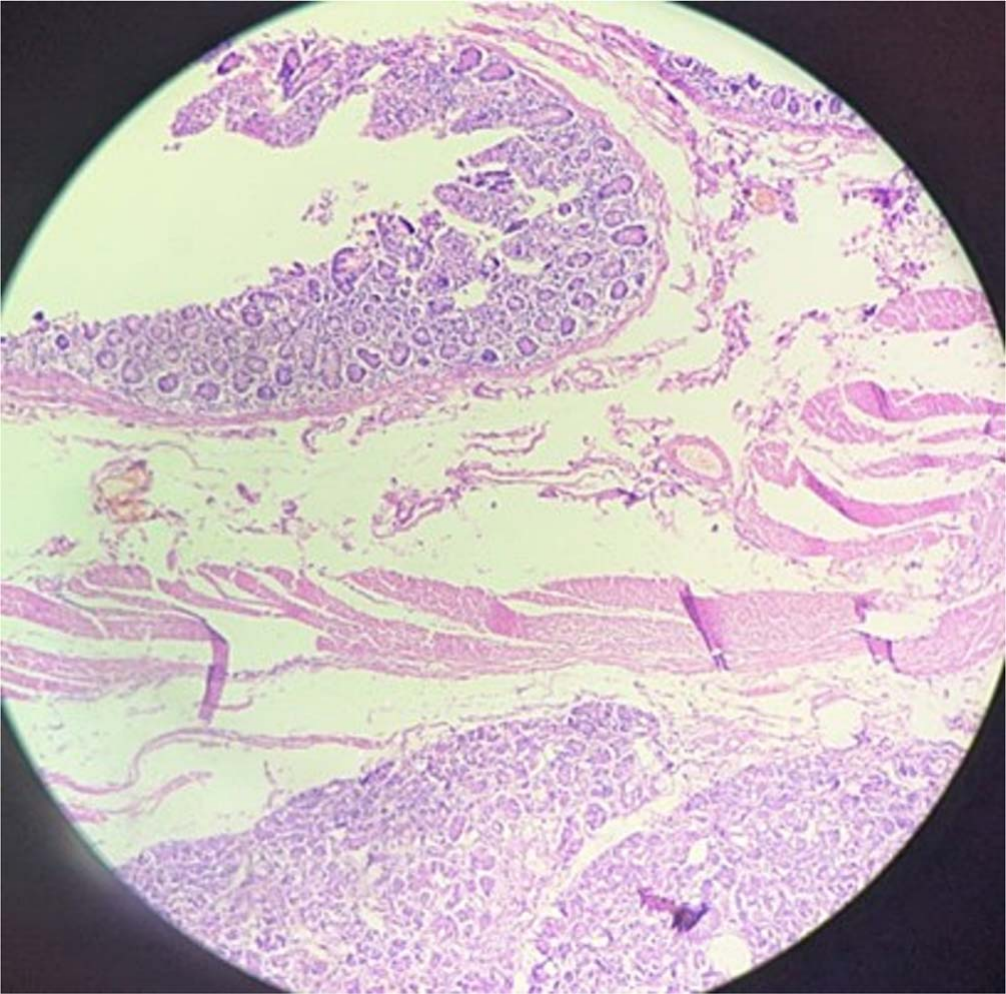
Histopathology of resected mass showing ectopic pancreatic tissue with pancreatic acini, islets of Langerhans, and dilated ducts in the submucosa and muscularis.

The histopathology report described a resected segment of the jejunum with a sessile polypoid mass (4 × 3 cm) showing ulceration and gray–white speckled areas. Microscopically, the mass contained ectopic pancreatic tissue with pancreatic acini, islets of Langerhans, and dilated ducts in the submucosa and muscularis. There were features of acute-on-chronic inflammation. No evidence of malignancy was noted (Fig. 3). The findings suggest pancreatic heterotopia with associated non-specific enteritis.

**Figure 4:**
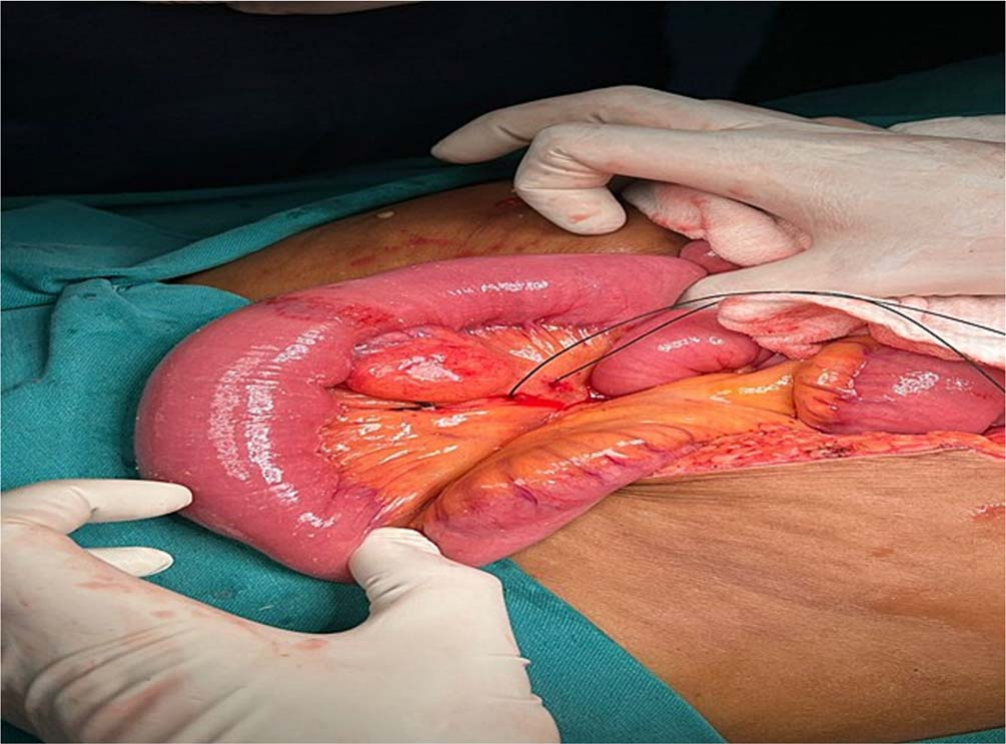
Intraoperatively, a firm, nodular lesion was identified arising from the jejunal wall. The lesion was submucosal in origin and well circumscribed without any evidence of infiltration into adjacent structures. Surrounding jejunal loops appeared normal without features of obstruction, ischemia, or perforation.

A segmental resection of the affected jejunum was performed by ligating and dividing the diseased segment’s mesenteric vessel and involved segment was excised (Fig. 4), and a tension-free end-to-end anastomosis was created using stapled technique to restore bowel continuity.

Finally, the mesenteric defect was closed to prevent internal herniation. The patient had an uneventful postoperative recovery with dietary and glycemic control advice provide at discharge. At follow up, she remained asymptomatic with normal bowel habits.

## Discussion

This case highlights ectopic pancreas’s rare symptomatic jejunal presentation, challenging assumptions that small bowel obstructions stem solely from adhesions or malignancy. Preoperative imaging mimicked malignancy due to asymmetric thickening, luminal narrowing, and lymphadenopathy, underscoring histopathology’s pivotal role in diagnosis. Type I heterotopia (acini, ducts, islets) infiltrating the muscularis propria mechanistically linked to obstruction, a finding seldom reported. Despite comorbidities, surgical resection achieved cure, affirming its efficacy in anatomically complex cases. The jejunum’s involvement—fewer than 10% of ectopic pancreas—and symptomatic obstruction emphasize its under recognized clinical significance. This report advocates interdisciplinary vigilance for ectopic pancreas in atypical obstructions, ensuring timely intervention and dispelling its dismissal as an incidental finding.

There are various clinical manifestations of symptomatic ectopic pancreatic tissue, including abdominal pain, nausea, vomiting, weight loss, melena, and consequently, anemia and anorexia [4, 5]. The definitive diagnosis is only achievable via histopathology which includes ruling out any malignant entity [4]. If the muscularis layer is involved, then persistent vomiting and dysmotility may be expected [6]. There are three types of ectopic pancreas described in 1909. Type I composed of acini, ducts and endocrine islet cells. Type II consists of acini and ducts without islet cells, whereas type III composed of only pancreatic ducts without islet cells and acini. Type IV was added later and it includes only pancreatic islet cells on histopathological exam [7]. The case we present is of Type I.

## Conclusion

This case demonstrates a rare symptomatic jejunal ectopic pancreas presenting as intestinal obstruction, highlighting the diagnostic challenge posed by its nonspecific symptoms. Definitive diagnosis relied on histopathology, revealing Type I heterotopic pancreatic tissue causing luminal narrowing. Surgical resection with primary anastomosis led to complete resolution, highlighting the importance of considering ectopic pancreas in unexplained small bowel obstructions. Increased awareness of this entity may improve preoperative suspicion and guide timely intervention, particularly when imaging suggests segmental thickening without malignancy. This report contributes to the limited literature on symptomatic jejunal ectopic pancreas, reinforcing the need for histopathological confirmation and surgical management in obstructive cases.
